# Care and Treatment of Old People1Read before the Medical Union, June 20, 1895.

**Published:** 1895-09

**Authors:** Samuel G. Dorr

**Affiliations:** Buffalo


### CARE AND TREATMENT OF OLD PEOPLE.[1]

By SAMUEL G. DORR, M. D., Buffalo.

THERE are several important practical considerations connected with age, be it either youth, infancy or old age. I will speak, however, of old age—that time beyond fifty.

As our bodies approach close to this half-century mark, congestion and slow degeneration of the tissues of important organs take the place of inflammation. After fifty, the body begins to show signs of loss of power and sluggishness of function, which is but the prelude to that sure, although it may be slow, decay, the progress of which is indicated by diminished sensibility, impaired memory, muscular weakness, scanty secretions, calculous affections, osseous deposits and organic visceral conditions. The death-rate statistics show that at fifty-five, three-fourths of the population have passed from the face of the earth. During the next thirty

1. Read before the Medical Union, June 20, 1895.

years, which is the period of life from fifty-five to eighty-five, there is a death-rate of twenty-three in a hundred, and this leaves just two out of a hundred who live beyond eighty-five years. A strange fact comes in here, and that is that the middle ten years of this last-mentioned thirty years, which is the period from sixty-five to seventy-five, is most fatal.

In fevers of old people, the mortality is five times greater at sixty than it is at ten, and four times greater than it is at twenty years of age. Under Care and Treatment of Old People comes the consideration of two very distinct classifications. First, that peculiar to the individual, such as heredity, diathesis, temperament, sex, age, and the like. Second, those things external to the individual and constituting the environment, like air and climate, place of abode, food, water, occupation, habits and mode of life. In these last things the doctor who treats and cares for old people must find his success, if he finds any success at all. Medicines and prescriptions will avail but little in lengthening life beyond the average where the environment and habits of the patient are wrong.

It has seemed to me that the atmosphere, with its heat and cold, its dampness and dryness, its dust, smoke and other conditions, has on the patient a power for good and evil little thought of by our general practitioner.

To advise removal from one state to another is not always possible, but to advise removal from one room to another, and being in the right heat and circulation of air, will always be practicable. When we contemplate the large amount of surface exposed by our bodies to the air and atmospheric conditions, we will at once recognise the importance of proper conditions, such as purity, temperature, humidity, and the like. The respiratory organs and air passages, made up as they are of nose, throat, larynx, trachia, bronchial tubes, bronchioles and air vesicles, about which is looped and massed the capillaries of the pulmonary circulation involving the action of the right side of the heart and the aeration of the blood, we are, at least, interested ; but when we calculate the effect of atmospheric changes on the skin, that great organ in which we must live, with its glands and nerves, and its capillaries, forming a large section of the terminating loops of the circulation from the left side of the heart, we must be convinced of the importance to longevity of proper atmospheric conditions.

The skin and the lungs do not degenerate and give out purely by reason of age, as do the eyes, the ears, the teeth and the circulatory system; therefore, if the being were never exposed to anything but pure air in a perfect climate, these organs would remain useful far beyond the average limit of life. If good air is conducive to length of life, the place of abode is proven to be a valuable factor in treatment.

Food and water, like the air we breathe, must be suited to our wants. We cannot dismiss these by saying they must be pure food and pure water. The fact is, they must be adapted to the wants of the patient, the same as medicinal substances. They may in the past have produced disease in the patient, although quite pure. The term pure cannot be made applicable to food and water, as many patients would be improved by water which contained some foreign substance; or food having that which is required by the body for its preservation. While we look for pure air, we must not look for pure water and pure food alone, but rather we must look for the water and food to contain that which is required by our patients.

In the aged, the teeth are frequently absent and the stomach is weak, hence difficulty in mastication and digestion; and with digestive difficulties come a large number of other difficulties. When the old, weak stomach is given too large or too difficult a task to perform, the sympathetic system of nerves is deranged fearfully, and life is frequently cut short by apoplexy or acute disease. When too large an amount of food or improper food is put into the stomach, too much for perfect digestion, there must be produced a vicious product in the system as a result. If the body, by reason of age, is unable to digest freely and perfectly a given amount of food, it is quite reasonable to suppose that the elimination of these vicious products would, by reason of the same age and consequent weakness, be very much delayed. For the above reason, it seems to me that elimination by all the organs of the body, not forgetting the skin, is quite imperative.

So far as water and liquids are concerned, I find myself forced to the conclusion that far too much is contained in the average individual. The circulatory system is under too great a strain, by reason of its fulness. Every pulsation of the heart produces an over-strain, and the elasticity of all the vessels composing the circulatory system is too early in life destroyed through mechanical effect, produced by a too large volume of blood in front of each pulsation.

Next in order mentioned comes occupation, and if food and water must be adapted to the wants of our patients, none the less must their occupation be suited to their peculiarities ; and it is not to be forgotten that aged people are quite likely to have peculiarities. There are those deep thinkers, like Gladstone, Bismarck and others, who must have hard mental work late in life. Reasonably protracted muscular work will bring sleep and act as a sedative very refreshing to nature. A perfectly selected occupation becomes an amusement which engrosses all attention and distracts all thoughts from other subjects. A few minds and a few bodies have to lie inert, they being worn to exhaustion. I recollect how intelligent was the mind of Mrs. Fillmore at the age of 106, when the body was weakened to that extent which prevented largely the use of the extremities. In her we have the example of the possibility of the brain outlasting the muscle, if cared for. Occupation of the muscles and the mind suited to the individual is conducive to longevity, while occupation unsuited to the individual is the reverse.

Habit, that wonderful plan of our nature, which is but the repetition of sensations, thoughts or movements, which are at first distinct, and may have been difficult, although voluntary, at last transfers us from the domain of attention to the domain of association ; and when the influence of association becomes predominant, as it will in most characters after long years of repetition, then the individual is a slave to habit. *He can no longer enjoy new sensations, new thoughts or new movements.* Every year of our lives places the yoke of habit more heavily upon our necks. The lives of old people are frequently cut short by habits which are persisted in with a full knowledge of their dangerous effect. Of all things on this earth and under the heavens, so far as the knowledge of man goes, habit is the most powerful influence for good or for bad which affects advanced life.

Old age and bad habits do not go together. Old age can only be to them who have no bad habits, or who have sense and will sufficient to correct them ; or who as perpetual youths have never let their sensations, thoughts or movements run in the rut of association, but have always held them under their attention and subject to the dictates of a wise judgment.

When your patient cannot resist a dangerous habit, and when there is no help or influence in the family of the patient to assist him or her to resist that which leads to destruction, then, as medical men, you may enter a prognosis of failure in your attempt to lengthen life beyond the average. There is no occasion for me to mention a long list of bad habits in this paper, but, as a fact of personal observation in my practice, I have to state that coffee, beer and tobacco are sending to the grave a fearful amount of people who are too young to die by natural causes.

Habits of one kind or another are in constant warfare upon every organ or system of our anatomies. The divine teachers tell us that habit carries her victories beyond this terrestrial sphere and destroys the soul, thus robbing us of both the future and the present. Some writer has said that the length of life is the duration of our arteries; and, as I think more and more on this assertion, I am impressed with its implied truth. The pulsations of the heart is the water hammer, which, by repeated blows, finally destroys the structure, thus constituting the physiological duration of our existence.

The heart must beat, and these beats are caused to deviate more or less from the standard of perfection by mode of life and habits of the individual. The heart, like the skin, brain and lungs, escapes the failure due purely to advanced age. The nutrition of the heart is especially provided for, and you always find the normal heart of old people hypertrophied. Some writers have made the error of stating this proposition in the light that old people have attained to old age by reason of having been possessed of strong hearts. The hypertrophy of the heart is physiological, whereas the alterations in the arterial coats whereby they lose their elasticity is pathological, and may, and may not, be the cause of the hypertrophy and dilatation in the heart.

There is so much to be said on this subject that it is best to stop all except very practical questions in this paper. Over-indulgence in food induces plethora, corpulence and loads the tissues with fat, which is a most dangerous condition for old people. Gluttony and the abuse of stimulants sends many to the grave. Prolonged exertion, violent emotions, petty troubles are daily causes of death. Gouty conditions and irregularity of heart's action are difficulties arising largely from faulty nutrition and defective elimination. The food of old patients with irregular heart's action had better be strictly regulated and persistently adhered to. I would recommend for breakfast, dry toast, a cup of tea with cream and sugar, and nothing else. For dinner, a little lean mutton, or chicken, or fish, dry toast, but no vegetables; a little brandy, tea or hot water. For supper, dry toast, fish boiled, tea or hot water. At bedtime, four or five ounces of hot water. This diet is for extreme cases of heart weakness, and when associciated with proper medicinal agents will seldom fail in its purpose.

The hasty conclusion of this paper has been brought on by the feeling that it is now too long.

300 JEFFERSON STREET.

